# Wearables for Integrative Performance and Tactic Analyses: Opportunities, Challenges, and Future Directions

**DOI:** 10.3390/ijerph17010059

**Published:** 2019-12-19

**Authors:** Jonas Lutz, Daniel Memmert, Dominik Raabe, Rolf Dornberger, Lars Donath

**Affiliations:** 1 Institute for Information Systems, University of Applied Sciences and Arts Northwestern Switzerland, Peter Merian-Strasse, 86 4052 Basel, Switzerland; rolf.dornberger@fhnw.ch; 2 Institute of Exercise Training and Sport Informatics, German Sport University Cologne, 50933 Cologne, Germany; d.memmert@dshs-koeln.de (D.M.); dominik.raabe92@gmail.com (D.R.); l.donath@dshs-koeln.de (L.D.)

**Keywords:** wearables, team sports, load monitoring, tactic, analysis, spatiotemporal data

## Abstract

Micro-electromechanical systems (MEMS) have reduced drastically in size, cost, and power consumption, while improving accuracy. The combination of different sensor technologies is considered a promising step in the monitoring of athletes. Those “wearables” enable the capturing of relevant physiological and tactical information in individual and team sports and thus replacing subjective, time-consuming and qualitative methods with objective, quantitative ones. Prior studies mainly comprised sports categories such as: targeting sports, batting and fielding games as well as net and wall games, focusing on the detection of individual, non-locomotive movements. The increasing capabilities of wearables allow for more complex and integrative analysis expanding research into the last category: invasion sports. Such holistic approaches allow the derivation of metrics, estimation of physical conditions and the analysis of team strategic behavior, accompanied by integrative knowledge gains in technical, tactical, physical, and mental aspects of a sport. However, prior and current researchers find the precise measurement of the actual movement within highly dynamic and non-linear movement difficult. Thus, the present article showcases an overview of the environments in which the wearables are employed. It elaborates their use in individual as well as team-related performance analyses with a special focus on reliability and validity, challenges, and future directions.

## 1. Introduction

Micro-electromechanical systems (MEMS) have continuously improved over the last two decades. Presently they are included in consumer products commonly referred to as “wearables” and offer data of sensor systems such as the Global Positioning System (GPS ) [1], accelerometers [2] and heart rate monitoring sensors [3]. This allows sports scientists to automate the time–motion analysis and therefore replace error-prone and subjective qualitative with quantitative data-gathering procedures in team and individual sports.

The quantification of team sports, in comparison to single-activity tracking, remains more complex as it evolves around the concept of two parties trying to outperform one another and win a competitive game sharing mutual objectives [4,5]. This is emphasized by the fact that during a competition there are constant activities in the form of actions and reactions to the movements of every participant. The constant adaption frames the whole contest as a product of dynamic interaction processes [5]. Hence, it is also required to track the off-the-ball movements of players to capture every dimension of these interaction processes. This highly dynamic nature of contests arises by chance, non-linear actions, such as short sprints and high-intensity runs, and other stress factors, especially game climax [5,6,7]. Specifically, the climax influences players in their decision-making and thus general performance [8]. The recent state of the wearables enabled scientists for more objective, sophisticated, and instant quantification in team sports [9]. The collected game data allows the analysis of physical [10] as well as individual and team-tactical behavior [11,12]. However, due to its complexity, the scientific analyses of tactical performance are still underrepresented [13,14]. Furthermore, the research focus remains in the area of individual physical performance [15]. Other researchers have recently begun to assess the performance of an individual as part of a collective formation as well as trying to quantify team performance. These studies encompassed the geometric average of a team by calculating formation team centroids [16], the lateral stretch of a team as well as length-per-width ratio [17,18], and inter-team distance (distance between centroids) [19].

This study provides detailed insight into the methods and the environments of performance analysis in the second section. This is followed by the state of wearables and their distinct technological components in the third section. This passage explains how wearables are replacing qualitative methods of observation, thus providing details on the level of data-gathering process. The fourth section comprises the individual performance metrics including a detailed explanation of each of these metrics and their current usage in elite organizations. This chapter showcases the application of the gathered data in the form of individual movement identification and quantification thereof, in the form of external and internal performance metrics. The fifth section elaborates the current measurements of tactics in game sports. Finally, the study is concluded with a discussion encompassing the challenges, opportunities, and future direction of wearables.

## 2. Environments and Methodologies of Physical Performance Assessment in Team Sports

To gain a deeper understanding of performance analyses, it is required to know the nature as well as the environment in which performance is conducted. In essence, a competition or championship contest in team sports as defined by [20] consists of “two parties (teams, doubles, or singles) that interact dynamically in order to score a goal/point and simultaneously to prevent the opponent from scoring”. A competition comprises elements of physical excellence, but also strategic efficiency, tactical efficiency, and specific perceptual skills [21] which can be summarized by the term decision-making [22]. However, these aspects are not isolated. A study provided insight that activity patterns of players and thus performance depend on the tactical issues of a competition [23]. Another distinct characteristic for team sports is the off-ball movement. In individual sports, the interaction always revolves around perfecting the action and manipulation with a game tool (e.g., javelin, tennis ball), whereas in team sports the off-ball movement is crucial to counter an opponent’s tactics. In a competition, teams do not possess detailed strategic or tactical information about the opponent, this is known as the phenomenon of incomplete and imperfect information [24] and thus the decision-making during a competition is based on subjective game observations by coaches and players. Furthermore, competitions contain elements such as chance, non-linear actions, and climax during the game [5,6,9,25,26]. These elements contribute to the dynamic nature of a competitive environment. This results among other factors, in subjective decision-making, mental and physical fatigue, influencing variability in data in between as well as within matches for performance indicators [5].

To increase the likelihood of winning competitions in such a dynamic game environment, every party prepares through practice. These improve the players’ performance by acquiring technical, physical, and psychological expertise with methods of deliberate repetition [27]. Another crucial objective of practice is injury prevention to minimize the risk of negative consequences from an excessive or inappropriate training load [15]. Therefore, objective measurement and analysis are substantial to comprehend the performance [28] and assess the effectiveness of training programs [29,30].

The coupling of competition, its analysis, and training includes interpretive tasks, which lead authors to employ qualitative methodologies in both the spectrums of environment [5]. The most commonly used methods were notational, physiological and video analysis, which are all indirect or delayed methods of quantification [31,32]. The notational analysis, is the process which relies mainly on questionnaires, diaries, and observations, whereas physiological analysis relies on standardized tests, and video analysis, which is an observational task after a competition has finished. Another study attempted to summarize the common practices. The authors defined a model including standardized tests and statistics from competition as quantitative measures. This resulted in a rating in standardized or natural settings as qualitative instruments [33]. The quantitative measures include perceived exertion scales, oxygen consumption tests, and the observation of live competition. All these qualitative and quantitative tests have downsides; they lack reliability, accuracy and while others are error prone [31,32]. Qualitative tests in particular are usually lengthy because of direct observation, limiting pre-match preparations to the study of only a few previous matches [34]. Therefore, they inherently suffer from subjective perceptions by the analyst. There are more objective analysis methods, specifically force plate and three-dimensional motion analysis, which take place in isolated laboratory environments. However, as introduced, locomotor and non-locomotor motion are only one part of the team performance in team sports and other influences such as chance and especially climax cannot be reproduced in laboratory settings.

In conclusion, there are two separate spectrums of performance environments: structured practices and dynamic, competitive environments. Competition consists of an interaction between two opponents that take advantage of one another to win, which is highly dynamic in nature. Practice, on the other hand, serves as a tool to refine motor-like psychological skills, particularly decision-making, and improves physical conditioning, employing methods such as approximation of game situations in the form of deliberate training. Thus, competitions exhibit non-linear and chaotic characteristics, whereas practices are conducted in an organized manner, with the limitation of only approximately reproducing a competitive environment in restricted training settings. Hence, the objective assessment of both environments is considered extremely difficult, as the qualitative approaches are biased and the quantitative methods do not represent the dynamic nature of team sports [30,35,36].

## 3. Sensor Technology and Data Gathering

The method of notational analysis builds upon the qualitative methods of observation. In the past, it consisted of handwritten motion formalization and thus allowed for quantitative analysis [37]. The current trend of automation of these notational procedures, executed by information technologies, is widely considered unbiased because the data stems from the original movement itself [38,39]. This chapter focuses on the first step in automated analysis: the data gathering. It introduces all the common sensors available, comprising a brief technical description, its application in general as well as specifically conducted research studies. Validity, reliability, and shortcomings of each technology, are discussed, with a look at the current established market products.

- Positioning System
  There are two types of positioning systems: the Global Positioning System (GPS) and the Local Positioning System (LPS). Both devices basically offer raw signal data, a timestamp with geographic coordinates, and a first data aggregation level consisting of velocity, distance and acceleration.
  The GPS is a tracking device that accesses the signal of the GPS satellites (or similar satellite networks such as GLONASS, GNSS, BDS, or NAVIC) networks to triangulate its own position [40]. The GPS precision of the raw signal data can be assessed by the amount of satellites connected to a device [40]. A more detailed approach is the horizontal dilution of precision (HDOP), which scores the geometrical spread of the satellites with which the device is connected. However, few manufacturers share this information in their devices output data [41]. Furthermore, the quality of the signal is highly sensitive to local interferences that can impede GPS devices capabilities to connect to satellites, such as large stadia. Finally, indoor localization is not possible at all [42]. Over the past decade, technological advancements raised its frequency of sampling from 5 up to 18 Hz [1] and it is “the most effective and time-efficient for monitoring workload within the team sport environment” [26]. Several studies found a general tendency towards a higher accuracy with increasing frequency values [7,43,44]. More recent studies confirmed these findings, yet expounded that non-linear motions, as they occur in most team sports, may decrease reliability and validity [26,43,44,45,46,47,48,49]. Especially two studies highlighted the decreased accuracy of the GPS devices during unstructured movements [26] and sprints at 5 m length [49]. In the earlier study the measured GPS metrics of a circuit “underreported the total distance covered (10–28%) [...] during random, unstructured court-based field-based team sports drills” [26]. This study was conducted with each athlete wearing two 5 Hz and 10 Hz and two 15 Hz GPS devices. The athletes completed a set of short distance movement drills of fixed 2 m, 4 m shuttle runs, and random sprint patterns to simulate real game movement patterns, among others. Similar results in comparable conditions were reported in an another study, even though the shortest sprints were slightly longer with 5 m [49]. The GPS of 18 Hz and 10 Hz still only achieved moderate and poor reliability respectively at this slightly longer range of 5 m, which is a rather long consistent sprinting distance for invasion sports environment. Furthermore, two of these studies even concluded that the 15 Hz are unexpectedly not superior to the 10 Hz devices [26,50]. Simultaneously other studies came to similar conclusions that increase in frequency does not always translate into higher precision [51,52]. One study found a possible explanation, that this is likely due to the sampling rate of the 15 Hz GPS is just an up sample via linear interpolation from a 5 Hz [53]. However, the latest study showed a 18.18 Hz GPS (EXELIO srl, GPEXE PRO, version M03, Udine, Italy) in comparison to the established 10 Hz GPS (Innovations, MinimaxX S4, version 6.71, Melbourne, Australia) achieving an overall higher validity and reliability in total distance and sprint mechanical measures [49]. The study comprised a circuit based on a variety of team-sport-movements. Each athlete wore an established 10 Hz GPS, and a newly released 18Hz GPS as well as a 20 Hz LPS device. The measures of the study were distance measures for each circuit section, the full circuit distance as well as mechanical sprint properties. The study concluded that the 18 Hz GPS in comparison to the 10 Hz provides an overall higher validity and reliability in plain distance and sprint mechanical measures [49]. The authors explained their findings with another study they conducted: the applied 18 Hz devices employ a true 18 Hz sampling rate [49,54].
  In addition to these hardware-related, raw data issues, there are also concerns associated with software processed data. Because of possible low-quality data, every available GPS has algorithms in place to correct outliers and filter noise. The issue at hand is not the filtering, but that the manufacturers’ techniques are mostly unknown to the research community, making replication difficult [41]. A further issue with the software is that commercially available devices undergo constant updates, which change basic processing algorithms [55]. It is, therefore, recommended not to update the devices during a longitudinal study, or find alternate ways to interpret the generated raw data as the interpretation of data may vary from a newer to an older version [41]. Another issue is the error-prone inter-unit reliability across different models and brands [7,44,56,57,58]. Thus, it is recommended that during a longitudinal study every athlete should receive a dedicated unit [44].
  The LPS on the other hand base their triangulation functionality on radio-frequency-technology paired with local antenna stations. These devices can support increased sampling frequencies up to 100 Hz or even 1000 Hz (see Table 1). This potentially allows for higher precision in terms of the detection of positioning in static and dynamic settings [59] and the identification of movement patterns [60]. The latest comparability study of GPS vs. LPS found that 20 Hz LPS (KINEXON Precision Technologies, KINEXON ONE, version 1.0, Munich, Germany) in comparison to the 18 Hz GPS overall shows superior validity and reliability in plain distance and sprint mechanical measures [49] Conversely, another finding of this study was that the particular version of the LPS also measured plenty of noise during standing, which was to be expected due to the higher frequency. The same phenomenon occurred while comparing 18 Hz vs. 10 Hz GPS devices. However, while comparing LPS to GPS the effect size was very large (d = 2.4–3.6), whereas the GPS comparison effect size was small (d = 0.3). In addition the LPS had higher occurrences of outliers based on measurement errors. However, the study concluded the use of latest GPS and LPS in practical application is still limited at this time [49].
  An advantage of this technology in comparison to the GPS is its versatility: for outdoor, indoor tracking as well as application in large stadia [51]. The continuous miniaturization could possibly enable ball tracking [61]. And lastly, building on the increased accuracy and the potential ball tracking, more precise tactical analysis is likely [62]. Conversely the LPS installation and calibration of said local base stations constitute a higher financial investment as well as decreased flexibility, because of the increased technical knowledge hurdles for setup and maintenance [60]. Due to these restrictions, only two of the commercially available devices contain LPS functionality.
- Accelerometer
  The accelerometer measures physical manifestations of force that affects the device such as acceleration and deceleration in uni- or multi-axial movements. The many peculiarities of the accelerometer differ in characteristics and application practicability [63]. After being optimized for automotive applications, further miniaturization allowed an adaption to other sectors, examples of which are biomedical and consumer electronics. Earlier studies described the devices as reliable [63] with continuous accuracy improvements [64]. The accelerometer samples multiple different dimensions of data: the actual x-, y- and z-axial forces with a time stamp as well as further processed data. The data displayed a lack of accuracy in the past especially in the area of complex, dynamic motions [65,66,67,68,69]. Today’s wearable devices mostly contain tri-axial tracking with a sampling frequency of 100 Hz. However, some offer sampling frequencies of up to 1 kHz, but still provide data at 100 Hz (see Table 1). Achieving this kind of maturity enabled the use of these devices in human motion analysis [2,9]. Later studies claimed the acceptable levels of intra- and inter-unit reliability [10,50,52]. The intra-unit reliability was confirmed in the most recent study, which took place in a mechanical test setting [70]. The test consisted of 19 devices of the type Catapult OptimEye S5 (Catapult Sprint Version 5.1.7, Melbourne, Australia, firmware Version 7.17), which were mounted on an electrodynamic shaker table. This shaker is viewed as a gold standard in environmental testing of electronic instruments such as NASA microsatellites. Alongside the OptimEye S5 devices, three single-axis reference accelerometers were strapped on the shaker table. Conversely to the positive intra-unit reliability, this study showed “trivial to extreme” variability in force measurements as well as approximated load calculations in terms of inter-unit reliability. Furthermore, the study showed a “mixed accuracy” when comparing the results, to a reference accelerometer and transforming the raw data to aggregated states. The resulting accuracy discrepancy ranges from small to trivial, therefore concluding that “the metrics [...] may be unreliable, especially when used to assess unpredictable multiplane, high-intensity actions” [70], which are prevalent in a team sports environment. Another recent study compared the validity and inter-unit variability of two Apex devices of 10 Hz (version 2.0.2.4) and 18 Hz (version 5.0) to measure the total distance covered of a 128.5 m circuit, 400 m trial and 20 m trial [71]. The authors did not find any meaningful difference between the devices in any of the measured distances. Nonetheless, the authors still found that “the data [...] has lower accuracy during high-intensity short distance activities than long distance trials”. The report cautions especially from direction changes of 180. as this “highly affects accuracy”. Both studies used methods of a circuit, which is built to approximate game or practice movement properties including different speeds on different lengths, change of directions and stationary waiting periods. However, they clearly differ from the practice or game environment described in Section 2. Thus, the argument of [30] that an appropriate methodology to quantify movement is currently missing, might still hold true. Moreover, the identified issue of missing reliability still consists of latest tests [70,71]. As mentioned quick and explosive movements are ubiquitous in the types of invasion sports. As the derived metrics, which follow in Section 4.2, heavily depend on the precision and reliability of the original data. Brands constantly innovate their methods, as well as algorithms to gather, and aggregate data to create a deeper understanding for their consumer. Thus, as with the GPS devices, it is difficult to compare results to previous versions as well as other brands [72]. Furthermore, many of the previously mentioned authors ask to communicate the algorithms employed in the devices to the scientific community to facilitate the replication and ongoing evaluation by independent groups.
- Gyroscope
  The gyroscope is a device to measure the angular rate and rotational velocity, which, when implemented, allows the tracking of angular velocity or the rotation of a body [73]. There are several types of gyroscopes: the most relevant ones for human movement tracking are the MEMS implementations, which employ vibrating mechanical actuators to sense the angular velocity [74]. Most commonly, the devices use electrostatic, electromagnetic, or piezoelectric actuating mechanisms to let the mechanical actuators vibrate and employ capacitive, piezoresistive, or piezoelectric detection mechanisms to sense the motion [63]. In the presented body of research, there is a common understanding that the main purpose of these devices is the automotive and navigation market and for that purpose, they are accurate enough. The application in sports has its own purpose: in sport areas such as skating, snowboarding, platform diving or gymnastics this device can accomplish what its designed for: measuring angular rates and rotational velocity. In comparison to the previously mentioned sport application areas, for a team sport participant it is rarely of advantage to spin, backflip or perform similar movements. Hence applying this technology in a team sport scenario, where the device is attached to the upper body, limits the device’s full potential; because the type of movements this device is designed to measure does not regularly occur in team sports [30]. A review of wearable microsensors did not mention specific shortcomings of a gyroscope [15]. However, the authors attested the gyroscope the ability to improve accelerometer-only data. As seen in the mentioned review, the gyroscopes are no longer an isolated measurement device. They are always coupled with an accelerometer and a GPS, thus recent, dedicated reliability or validity studies in exercise environment could not be found.
- Magnetometer
  A magnetometer gathers data about the magnetic field of the earth and its strength to derive the orientation direction of the device [73,75]. It is susceptible to local disturbances and other similar magnetic forces, which can significantly interfere with the orientation process and thus lower signal quality [76]. As some of the main team sports are conducted indoors, or in some sort of stadium, and the only information gathered is the spatial orientation, the magnetometer rarely finds an application by itself in load monitoring [30]. The results of another study indicate that by approximating magnetometer data, the gyroscope signals can be replaced [73]. As with the gyroscope, the magnetometer is coupled with a GPS and/or accelerometer and thus no recent and dedicated reliability or validation studies in a sport setting could be found.
- Heart Rate
  In comparison to the other sensors, the heart rate measurement is a tool to measure an internal body function, hence [77] named the group internal load monitoring tool, which will be discussed in Section 4. It has established itself as one of the most commonly employed tools to quantify practice intensities [3] as a non-invasive method [78]. It is commonly accepted as accurate [79]; however, as the tool tracks internal body functions it may be influenced by external factors such as temperature, humidity, altitude or internal ones such as the hydration status [3]. Despite the high accuracy findings, the authors stated that the heart rate generally displays slow response to changes in intensity and, therefore, valued it an inappropriate measuring tool to monitor performance intensity. However, there is contradicting evidence in [32], where the authors created an index system to be able to concretely interpret a training load. Nevertheless, the technology employed as a chest band is widely recognized. It is currently the only internal measurement device, which allows non-invasive, quantitative, and thus objective measurement [79]. Another study established assumptions that there is a linear relationship between heart rate and oxygen intake [77].
- Muscle Oxiometer
  The muscle oximeter is one of the latest technologies to reach the consumer market. The first products were, in relation to the other devices, only recently launched (2006). It is an internal measure to establish an understanding of oxygenation and hemodynamics in muscle tissue. These types of devices use near-infrared light and exploit their oxygen-dependent properties to determine oxygen saturation in muscles. The process of near-infrared spectroscopy (NIRS) calculates a weighted average of hemoglobin in a vascular bed and myoglobin in muscle fibers [80] (cf. [81,82]). Because of its novelty, the only review of scientific studies stated that the current quality of research is rather low, due to the low number of available studies and asks for further research to increase the state of knowledge [80]. A finding of the review is that NIRS data can be used as a robust marker. However, there are several drawbacks. One being the inaccuracy of the measuring surface of the device (a few cm^2^) providing sufficient data to derive muscle, or body load metrics. Another issue is the adipose tissue thickness influences the reflection of infrared lights [83]. Lastly, further elucidation is required in terms of relationships of muscle oxygenation, heart rate, and muscle activation [80].

All the previously mentioned sensor technologies have proven and documented strength and weaknesses. To address the previously outlined issues, researchers tried to combine all available sensors to exploit the strength of each and overcome their individual shortcomings [84,85]. This resulted in the launch of several sensor conglomerates under acronyms such as inertial motion sensor (IMS) or inertial measurement unit (IMU) [68]. The only current exception is the muscle oximeter, it is currently not included in consumer IMS. However, due to the Bluetooth connectivity of the latest devices, it is likely that the muscle oximeter will soon be part of a product suite. At the core of these IMS systems lies the attitude and heading reference system (AHRS) that joins data from accelerometers, gyroscopes, and magnetometers into an output quaternion that comprises drift-free and accurate orientation values [68,86,87,88,89]. In detail, the combination allows the estimation of an object’s orientation by applying a Kalman filter [90] or similar weighting algorithms [91]. Older studies collected in [68] attributed a higher error rate in orientation than claimed by the manufacturer, and further criticized that the tests were “limited to a single gait stride, a slow box lifting task, and a few activities of daily living”. Therefore, the author introduced their own testing of upper body movement and concluded that “quasistatic testing of the IMS against gold standard data demonstrated that manufacturer claims for rms errors are verified”. However, in complex locomotor movements the authors confirmed the findings of [92], namely that orientation errors tend to overshoot during periods of fast orientation changes. More recent studies confirmed these findings [45,55,70,93], which is worrying because exactly this kind of chaotic and unstructured movements is to be expected from team sports motion analysis [6]. Furthermore, one shortcoming that some of the above introduced technologies share is the noise of gathered data. On one hand the human movement can be cluttered with noise [94], on the other hand when the data is not accurate, the data shows movement even if there is none (e.g., imprecise GPS location while standing [49]). Hence, there are smoothing algorithms in place to remove this noise and compensate for inaccuracies in measurement. A common approach to cancel noise from human movement data is a low-pass filter [94]. This method comprises a cut-off of at a matching frequency, which varies in different types of movements [95]. Furthermore, if the cut-off frequency is too high, there still might be too much noise. On the other hand, if the cut-off is too low, there might be crucial data cut-off [94]. Another argument recently returned to the spotlight by [41] was the discrepancy of real-time data vs. post-processing, which was originally discovered in another study [28]. The study identified the devices’ technology as the main root for this error. Considering the advancements in sensor technology it is safe to assume that this matter has also improved. However, it is still recommended that further research in this area be conducted [41].

Currently the following commercially established devices are available (see Table 1). The table introduces these products as presented on their manufacturer’s website and includes the devices’ location sampling frequency (Loc. Freq.), location measurement accuracy (Loc. Acc.), sampling rates of other included sensor technologies, and whether it contains capabilities to track an athlete’s heart rate. Please note that the wearable market has experienced major mergers and acquisitions in the past five years. Several enterprises, such as GPSports and Prozone, have been acquired by the bigger companies. Hence, their products, which have been employed in the mentioned studies, are commercially no longer available. Furthermore, devices such as the Minimax S4 of Catapult Sports or SpiElite of GPSports, which were commonly employed in the mentioned studies, are outdated and replaced by newer versions of the respective product. Based on the versioning issue and the general novelty of the devices (or their software) there are rarely any reliability or validation studies. With the exception of the Catapult OptimEye s5 (Accelerometer Test, [70]), GPExePro + Kinexon One (GPS Test, [49]) and Apex (GPS Test, [71]). These studies were included in the respective technology section.

The application of these devices’ in elite sport clubs was surveyed in a study of 41 football (soccer) clubs around the world. The interviewees answered questions about their applied devices and metrics in their environment [48]. The questions evolved around the tracking systems in practice as well as competitions. The results show the deployment of utilities ranging from subjective ratings, such as coach observations, to accelerometers, with heart rate measurement, to time–motion analysis in practice. Clubs employing sets of technologies usually develop their own “club-specific calibration equations” to be able to merge data from different technologies [96]. However, in comparison to a practice session, the only tool employed to track players in a competition environment was the time–motion analysis, the others such as accelerometers and heart rate monitoring are not tracked in any of the 41 sports organizations’ competitions.

In summary automating the process of movement quantification is still in development. There are hardware and software issues with the individual sensors and their conglomerates. Conversely, the latest studies have shown promise for the detection of more complex and coherent movements for a wearable. This success is based on the increased frequency of up to 18 Hz for GPS and 20 Hz for LPS, which resulted in superior intra-unit accuracy and reliability. However, the devices’ data contained more noise and outliers. Furthermore, inter-unit reliability showed trivial to extreme variability in force measurement and load calculations. Hence, the latest conclusion is still that practical application is currently limited. A further issue discussed in the research community is the constantly changing versions of software and hardware, which: (i) mostly invalidates older studies, (ii) does not allow for a data archives as the data constantly changes. A rather abstract and non-documented issue is that in a competition in which wearables are used, a coach can only supervise his own team. An opposing team is unlikely to share their data, thus not allowing for the analysis of opposing players and thus creating a strategic advantage (e.g., substituting a player on a position, where the defending player is tired). If in a future case, where a league decides to share the team data to all partaking parties of a competition, there is still the real-time vs. post-processing dilemma. Nevertheless, it seems that the current situation with the wearables, does only allow for tracking in a practice environment. There teams work with a wide variety of systems, including automated video analysis. However, given the fact that the inter-unit reliability of MEMS trackers remains questionable, combining data from different technologies all together is an even greater challenge, especially with respect to team sports and tactical analyses.

## 4. Individual Analysis

In a recent study, a generic two-category approach to performance measurement in sports: internal and external monitoring was presented [77]. Internal load, refers to the aggregation of the physiological and psychological stimulation occurring during training activities, whereas the external load is defined by physical work completed by an athlete, namely the quantity, quality, and organization of a workout [97]. The measurement of the internal load requires a holistic approach to generate a comprehensive understanding of performance. It consists of three subgroups: cardiovascular and respiratory measurements, humoral parameters, and neuromuscular parameters [77]. Thus, the assessment of internal load requires multiple monitoring devices and methods while working out, undergoing invasive measurements. This not only compromises training procedures, but also the quality of data collection [77]. Because of the given complexity and invasiveness, researchers attempted to approximate these methods by exploiting external monitoring data from tracking systems, which resulted in the publication such as the player load [10]. The calculation of the external load on the other hand, bases its metrics on the objective quantification of movement, by means such as the GPS or accelerometer [98,99]. The recorded data is then exploited to classify movements, capture performance intensity, and derive further metrics, such as peak speed, speed zones, and sum of accelerations and decelerations. This chapter introduces the generated insight from the data gathered as elucidated in the previous chapter.

### 4.1. Movement Classification

The process of movement classification was conducted in a local, manual manner and was widely known as notational analysis. It consists of the identification and notation of all movements, its intensities and directions and other sport-specific activities in a given session [37]. The aggregation of this data allows the analysis of quantity, quality and organization of said session [97]. Therefore, the movement classification is purely an external load measurement. The introduction of wearables and time–motion analysis allowed scientists to start automating the process of data gathering, as elucidated in the previous chapter. With the application of classification algorithms on sensor data, researchers tried to fully automate the notational analysis by classifying a set of data points. This process encompassed supervised learning algorithms such as Naïve Bayesian, Gaussian Mixture Model, or Hidden Markov Models [100]. This replacement of manual, biased evaluation added objectivity to load quantity, quality and opens the field for potentially further metrics.

Of the four sports archetypes introduced by [101], namely target sports, net and wall games as well as field games seem to be very susceptible towards the MEMS technology in combination with movement detection. A systematic literature review by [15] accredited the wearables the “ability [...] to accurately detect sport-specific movements in a wide range of environments”. The review contains literature (*n* = 28) about the motion detection of team sports mainly comprises the detection (*n* = 19), analysis (*n* = 8) of non-locomotor sport-specific motions, and a combination thereof (*n* = 1). Out of these 28 studies, seven were considered team sports: four in some form of rugby (rugby, league, union and Australian rules football), two baseball, and one cricket. The research regarding rugby solely focused on detecting and quantifying the impact of tacklings and collisions. The two baseball studies investigated the throwing and swing of baseball players. Lastly the cricket study encompassed the detection of a specific throwing type (fast bowling). Another more recent inertial sensor study reviewed 286 publications [102]. One of the analyses separated the papers by sport, where multiple counts for comparative studies across sports were made. Out of all the papers analyzed using sensor data, only 74 (25.3%) investigated team sports. The reviews conducted during these studies comprised 20 studies in rugby, 12 in Australian football, ten in soccer, five in baseball, four in cricket, four in field hockey, four in volleyball, three in ice hockey, and leaving the final 12 distributed over several other sports. Out of these named 62 team sport studies, 24 are concerned with physical demands for a certain sport, 20 encompass detailed motion analyses of a pitch (baseball), slap shot (ice hockey), and ten discuss some sort of tackling forces (soccer, rugby). Therefore, a more abstract concern is the current state of research, where validity and reliability studies are mostly considered for simple locomotor tasks or non-locomotor sports. Another very recent study reviewed inertial sensors in the domain of combat sports [103]. Most of their studies they included recorded performance measurements on “striking quality, automatic classification of strikes, automatic scoring, head impacts, athlete endurance, power and mobility and grappling technique”. Their findings regarding the technology involved was positive. The proposition for future research was the fusion of different sensor data and merging of algorithms. As well as determining a consensus for optimal sensor positioning to increase data quality. This concern is already established in the scientific community. A study shows that less contact in sport enhances the likelihood for successful movement detection [45] and the identification of high dynamic motions, such as tackling, remains difficult [104]. Consequently, it is even unclear whether consecutive, dynamic movements in high intensities can be distinguished and thus classified [52,71]. However, this seems to be crucial since another study provided an estimation of over 900 different discrete movements. This includes motions from standing still to running in several intensities, forwards and backwards, sideways slide steps, jumping, and other soccer-related skills, such as kicking or passing the ball [105]. A review study of [106] summarized and updated the standard of eight modes of motion in time–motion investigations. The authors created the Bloomfield Movement Classification (BMC) focusing on invasion sports, which allows investigating speed agility quickness demands of sports. Such a movement classification builds the foundation for the next section. The ability to distinguish between a set of different activities and the ability to create a movement profile over a given practice or competitive session enables scientists for more detailed performance analysis.

### 4.2. Performance Metrics

The calculation of performance metrics applies the aggregated movement data from wearables to compute external and internal performance metrics. However, there are several methods of deriving such a metric. For example, to calculate the velocity and distance from GPS data there is the Doppler-shift and positional differentiation. As with the data gathering, the methods and algorithms are different for each brand, software and hardware version of wearables, and thus make it impossible to create a long-lasting, comparable dataset. However, in theory, all follow the same thought process of exploiting the external, non-invasively acquired data to gain insight into the players’ external performance and derive the internal load. These metrics are highly dependent on the accuracy of the gathered data to identify performance improvements over measurement errors [107]. In a consensus statement [108] estimated all measures, based on GPS, time–motion analysis, and accelerometry, validity as medium-high and their reliability as medium. Thus, the metrics inherently carry identical detriments. The following list contains the most common examples for the process of performance metric calculations (adapted from [48]):

- Distance
  Distances represent the total volume of distance covered in a measured session, whereas relative distances represent the relative volume of the distance per minute of a measured session. The recent 18 Hz GPS comparison study has shown that the latest commercially available wearables are good at calculating total distances in a reliable and valid fashion [49]. However, the study also showed that 5–10 m sprints are still only at a “moderate” validity level, with 20 m sprints barely making it to a “good” level in terms of validity. Reliability, on the other hand, measured a “good” level in every circuit section as well as overall circuit length. Given that most team sports mostly consist of such 5–20 m sprints, disregarding body contact and peak impacts, the validity of GPS in game data inherently can maximally be of “moderate” validity.
- Velocity
  Peak Speed is the highest speed in a measured session. Another metric is the number and distance of high-speed runs, this determines how much of the total volume was spent at a certain velocity, which is typically classified in “work-rate zones”. For the peak speed, as with the distances, the recent wearable comparison study has shown that a shorter distance leads to a lower validity in the overall distance covered. Thus, the validity of peak speed for short distances inherently suffers [49]. The work-rate zones allow making assertions such as to the time spent in each zone. However, the distribution range of speeds for each zone heavily depends on the examined sport [28,45,109]. Furthermore, certain researchers argue for individual, position-dependent zones, as players have different physical characteristics [110,111]. Another sensitive topic is the “minimum effort duration” [41]. This is a customizable setting, which defines how long an activity must last to be identified as such. For example, an activity is only labeled as running if the speed of the measured activity is over 18 km/h over a period of 0.5 s. Then, the minimum duration for the activity running is 0.5 s. This allows for filtering outliers, created by qualitatively bad GPS data. However, this setting is not consistent for all types of activities. Therefore, every brand, device, and sometimes even different versions of the same device have different minimum effort durations for certain movements. Hence, again increasing difficulty to compare results or create a data archives over time.
- Acceleration
  There are different measures and metrics for acceleration. One is the number of acceleration and deceleration accumulates the sum of accelerations and decelerations and classifies them into different zones according to the acceleration or deceleration value [45]. Another is the peak acceleration of an activity (m/s^2^).
- Number of impacts and collisions
  This metric counts the occurrences of impacts and classifies them according to impact zones, separated by impact strength valued in G-forces [45]. However, as with the distance of high-speed runs, the ranges are quite arbitrary and have not yet been scientifically validated. A study showed that the main limitation of this metric is the missing accuracy of the wearables in a highly dynamic motion [104]. As with the speed zones, these values might differ from one sport to another and the brands themselves decide on their arbitrary ranges.
- Stride analysis
  There are several forms of stride analysis. There are full-fledged solutions that include the average peak impact of each step on either foot (see Step Balance, STATSport). It is shown as a percentage of the total impact. An even distribution of 50% indicates efficient gait. On the other hand, an uneven distribution might indicate an overcompensation and further investigations might be suitable to find the root of the imbalance [112]. The other form of stride analysis are quantification of stride characteristics. One study investigated stride variables, such as contact time and flying time, as well as vertical stiffness [113]. The study highlighted the potential to assess this metric.
- Player Load
  This measure is supposed to unfold the total external mechanical stress accumulated during discrete game activities. The most commonly reported of these types of metrics is the player load itself [114,115]. The authors explain that the “omission would underestimate the rigor of competition”, because accelerations are energetically more demanding than constant velocity [116], and decelerations cause significant mechanical stress on the body [117]. The similar load calculation is the “body load” (GPSports), the “new body load” (GPSports) and the “dynamic stress load” (STAT Sports). They are the same as the player load, or a derivative thereof.
- Force Load
  This refers “a cumulative measurement that sums the forces produced from both foot strikes and collisions” [118]. The advantage compared to the body/player load is the ability to reflect locomotor-related impact and “provides better estimates of overall foot work and impulses” [96].
- Metabolic Power
  The aim of this metric is to calculate the total internal energy expenditure during training or competition [119]. The calculations in a test that contained linear running trials were documented as reliable [120] Another study testing in a confined circuit with 19 m length, with the focus on small sprints and change of directions came to different conclusion [121]. They outlined that “locomotor-derived metabolic power underestimated very largely the actual net metabolic demands of the drills”. A recent review study confirms these findings and elaborates that “recent research findings question the validity of this construct in the context of team-sport-specific movements” [96]. More so, the authors state that it is only an incomplete measure of the internal load and a too broad marker of the external load. The consensus statement of [108] evaluates the validity as low-medium and the reliability of the metabolic power as medium.

Albeit the validity and reliability concerns, practitioners are applying these metrics. In the previously mentioned survey study 41 elite football (soccer) clubs have answered questions about their applied tracking systems and metrics [48]. There the clubs valued the following top five metrics for practice training load: acceleration variables, total distance, distance covered with speeds greater than 5 m/s, Metabolic Power variables, and heart rate exertion, whereas in a competition the top five training load variables of interest were: total distance, distance covered with speeds greater than 5.5 m/s, distance covered with speeds greater than 7.0 m/s, acceleration variables, and average velocity. One of the main findings of the study was that “there is no universally adopted monitoring approach in high-level football” [48].

## 5. Measurements of Tactics in Game Sports

The outlined technical possibilities of [122] wearables in sports science and sports practice also promise advances in the field of tactical analysis of team sports. This is exemplified by the sport of football (also known as soccer), where the current development benefits scientific research [45]. In football, automated match analysis with the help of digital event and position data has recently become increasingly popular in science [14] as well as among practitioners [58,123]. Previously, hand annotated data provided the main source of data for statistical match analysis [124,125]. Although firmly integrated in practice, this method suffers the aforementioned crucial limitations of restricting pre-match preparations to a small number of games [34] and carries a subjective note by the analyst, which makes scientifically sound inter-match comparisons infeasible [5].

The recent proliferation of (semi-)automatically collected match data offers a promising alternative. Especially so-called position data, generated by player tracking technologies such as wearables, have recently become widely available, causing a boost of scientific publications on tactical analyses [11,12] —a field that has been long underrepresented in the scientific literature on performance analyses [13,14].

In addition to wearable technology, camera-based tracking technologies or combinations of both methods [51], offer another possibility to generate spatiotemporal data. Thus far, they have been the preferred data source for tactical analyses on 11-versus-11 game play and make up for the larger part of the scientific literature on the topic. This is due to two reasons: on one hand, wearable tracking devices have, until recently, not been allowed to be worn on the pitch by the players. On the other hand, the accuracy and validity of methods relying on global positioning seem until today insufficient for tactical considerations where absolute positions of the players are of great importance [58,126]. Yet, in 2015, the International Football Association Board principally allowed the usage of electronic player tracking systems (EPTS) [127]. Furthermore, local positioning systems seem to provide an adequate level of accuracy and have recently been successfully used in official matches [128]. Therefore, it can be expected that an increasing amount of data collected by wearables such as GPS [93] or radio frequency-based devices [59] from official matches will be available for scientific investigations in the future.

Nevertheless, position data has already proven to be valuable for tactical analyses in game sports. One important research field that regularly relies on wearable technology is the investigation of so-called small-sided games (SSGs). These experimental approaches are highly prominent in the literature, as they provide a popular training drill and research object. They resemble the normal game of 11-versus-11 football, yet are played by fewer players (often ranging between two and nine players a side plus possibly a goalkeeper) and with smaller pitch sizes. Additionally, rule modifications and task constraints allow manipulation of the game setting. As SSGs replicate several features of actual match scenarios in a reduced setting, it is believed that they are an effective tool to coach technical and tactical aspects of the game in a controlled environment with high repetition rates [129,130,131]. Some of these measures are now also being transferred to 11-versus-11 scenarios in experimental setups [122].

Research on SSGs usually involves field experiments, in which the effect of manipulating subjects (player’s age and expertise) and task constraints (pitch dimensions, number of players, numerical superiority/inferiority, additional scoring targets, winning/losing situations, introduction of additional free players known as “floaters” or “jokers”) on dependent variables is being examined. While dependent variables used to be mostly of physiological nature [131,132,133], researchers recently started to focus on tactical aspects as well. These are modeled mostly by geometric features such as team centroids, distances, and the surface area of players.

Subsequently, we highlight some of the findings focusing on tactical performance analysis where position data stem from global as well as local positioning systems, following a common distinction between individual-, group- and team-tactical measurements. As the units worn by the athletes can combine multiple sensors as highlighted above, these studies are equivalently often complemented by information such as aggregated load or heart rate measurements.

### 5.1. Individual-Tactical Teasurements

For the individual player, making the right decisions on the pitch is essential [134,135,136,137,138]. To improve decision-making abilities in adult and especially in youth players, it is believed that SSGs offer an effective tool for training [131].

One of the first studies comprising GPS data collection and focusing on tactical analysis compared approximate entropy (ApEn) values in a pre-post-test design [13]. Students with good technical, but low tactical ability were tested before and after taking football courses at a university. The results showed that the distance of players to the team centroid (geometric average of a team, compare also to [16]) was more regular after the 13-week training, suggesting that this trend models the acquisition of tactical expertise. To assess regularity, the authors used ApEn calculations [139], a method computing regularity in (non-linear) time-series data. In tactical performance analysis, this technique is employed to quantify regularity in patterns of a certain performance indicator over the course of a fixed time interval [19].

Differences between tactical expertise and decision-making in football on the individual level have furthermore been linked to differences in skill level [140,141] and age [142]. For example, one study showed that the lateral stretch of a team as well as length-per-width ratio differed for Under-17 and Under-19 elite male youth football players, suggesting that the older players displayed a better ability to use the width of the pitch [17,18]. Another factor in tactical performance and decision-making seems to be mental fatigue as shown by [8].

### 5.2. Group- and Team-Tactical Measurements

Likewise, players have to make good decisions and find effective strategies as a group to be successful. Using a local positioning system, researchers were able to categorize some of these strategic patterns in a 3-versus-2 SSG with spectral clustering [143]. Contrasting different SSG setups (2-a-side, 3-a-side, 4-a-side, and 5-a-side) and calculating ApEn values for team centroids, the distance between players and the centroid, and the inter-team distance (distance between centroids), more regularity arises the more players participate in a SSG, indicating a more structured organization within a team [19]. A subsequent analysis extends these results by adding numerical superiority or inferiority situations into the SSGs and different ability levels to study cooperation effects [141]. Another finding concluded that team dispersion, as calculated by the difference between players and the total game centroid (combination of both team centroids), differs when manipulating the number of players in SSGs while the distance to the closest opponent (TS, team separateness) stays constant [144]. Yet, team separateness seems to vary when introducing goalkeepers and floaters into the SSG [145].

One study also found differences in distances to team centroid when manipulating game status (winning or losing) and team unbalance (numerical superiority) in a 5-a-side SSG [146]. Another effect on inter-team distances and team centroids can be observed when playing SSGs with two or six scoring targets, respectively [147]. In terms of playing shape determined by the surface area or length-to-width ratio of a team’s positioning, different skill levels of players in SSGs also seem to have an impact [140].

In line with the dynamical systems approach, [148] investigated how the team centroid and the team surface area (the convex hull around all players of a team) change in a 4-a-side SSG played by young male elite players (LPM, 45 Hz). They found that the team centroid of two opposing teams is highly correlated in the forward-backward direction over the course of the game and noticed a specific pattern of crossing centroids in approximately half of the scored goals (*n* = 19) [148]. Investigating inter-team distances, and surface areas, [149] found that when manipulating pitch sizes in SSGs, shorter pitches lead to smaller longitudinal inter-team distances, narrower pitches lead to smaller lateral inter-team distances and a smaller playing area leads to smaller surface areas [140,150]. Further crossover effects indicate that teams adjust their lateral and longitudinal positioning to the playing conditions [149] and differences can be found between age groups [17,18].

With respect to group roles, one study calculated centroids for defenders, midfielders and attackers, respectively (4-3-3 formation), as well as absolute distance from players to each centroid in a regular 11-a-side setting [151]. ApEn measures accounted for regularity within the movement patterns and relative phase measurement quantified synchronicity in running directions of players. The results included a smaller distance of players to their own centroid together with a higher grade of coordination as measured by ApEn and relative phase variables [152]. Coupling was stronger for midfielders and weaker for attackers, which the authors link to their specific tasks during the game. A recent study by the same author has linked regularity in between-player-distances with passing networks in different age groups [142], whereas [153] investigated the connection between restrictions in individual playing space by field zones to game dynamics employing dynamic overlap analyses and passing networks.

Another stream of research closely linked to performance analysis is the linkage of match play demands or load to a player’s role or position in an 11-versus-11 setup. [128] compared high-intensity runs, sprints and turns between common player roles (e.g., center back, wide midfielder or forward) in official matchplay, combining the use of a local positioning system and a sensor system worn by the players comprising an accelerometer, a gyroscope, and a compass [128]. Similarly, [154]. investigated player roles in terms of distance covered, accelerations, and time spent at different heart rate intensities, noticing potential inaccuracies of the GPS system for high-intensity activities [154,155]. For the whole team, differences were found between common playing formations regarding total distance, high-speed accelerations, and decelerations as well as high metabolic load distance [156].

Overall, a rapidly growing body of literature can be observed that makes use of both local as well as global positioning systems and additional sensors such as accelerometers, gyroscopes, or heart rate monitors. Research has focused on small-sided games, yet, research on full 11-versus-11 game setups has caught up with more to be expected in the future. Caveats and limitations include the not entirely resolved accuracy problems, in particular for GPS and high-intensity activities [51,133,157]. Furthermore, the comfort of wearing the devices has also raised issues [28,123].

## 6. Discussion

The introduction of combined sensor technologies in the form of wearables is currently in the process of rendering old qualitative observations and other measurements obsolete. The new devices enable quantitative tracking and therefore allow the extraction of objective data for performance analysis. For this automated analysis procedure to achieve reliable and valid results, both data gathering and data processing require validation. With the most recent increase in frequency, the devices seem to show promise with respect to the data-gathering process. Nevertheless, the underlying assumption that if devices are worn in practice or game sessions and can reliably detect and quantify movement, validity is adhered as the tracking data stems from the activity itself. This is widely considered unbiased in all applied domains, hence the method of data collection is rendered valid. However, the conducted studies still raise questions, as the confined test settings are not comparable to diverse practice or competitive environments. Yet, the more delicate situation is the one of processed performance metrics. As the gathered data quality is still questionable, the metrics will inherently carry equal drawbacks. This negatively influences the practitioners’ ability to determine performance improvements over measurement errors. Calculations of the external load currently are considered more valid and reliable than calculations of the internal load. This is due to industry standard devices such as laser pistols that can be a criterion measure for peak speed with very high reliability and validity. However, this is more difficult for the case for internal loads, such as the player load or metabolic power. Furthermore, there are still discussions ongoing, what these internal load metrics eventually are supposed to assert. Albeit, it is important to note that these metrics require their own reliability and validity studies.

However, there is an undiscussed, but related issue: The manufacturers claim that the devices “minimize injuries”, “display intensity indicators”, “are highly accurate indoor and outdoor” as well as “powerful and small” and they generally state that the devices “help athletes and teams perform to their true potential” or “it will add value to decisions at all levels of performance sport”. The authors of this research doubt the correctness of these claims because the reliability of the metrics substantiating these statements are as stated a matter of data accuracy. Yet, the data they acquire are currently neither sufficiently reliable, nor valid, consequently their processed metrics cannot be either.

### 6.1. Challenges

Currently, the main challenge is the device itself. In soccer, FIFA adapted the rules and has now allowed equipping players with a tracking device during championship contests. However, in many other sports such as basketball and handball with plenty of body contact, where no protection gear is worn, the device is uncomfortable at best and prone to injure a player at worst. For example, in handball and in volleyball, the players constantly roll over backwards to absorb landing impacts. Furthermore, as previously discussed the wearables lack accuracy in highly dynamic motions. This restricts the methods of processing the accumulated data especially in the area of invasion sports, where this kind of erratic movement is common. The more dynamic and chaotic environment than in experiments, which is prevalent in practice and ubiquitous in game situations, quickly highlights the wearables’ limits. This is especially prominent in fast paced sports such as basketball or ice hockey with plenty of body contact and change of directions. Thus, it is unclear whether consecutive movements can indeed be detected and quantified with current technologies. Adding the lack of comparability to the level of inter-unit, -versions and -brands due to different methodologies and algorithms, results in a sub-optimal situation for practitioners. There, devices restrict individual performance analysis where precision is key to distinguish between performance improvement and measurement error. A fact that has not received much attention in the scientific community so far is the further loss of data accuracy when wearables are employed in real-time application in comparison to post-game analysis. IAnother issue brought forth by [158] are the resulting influence of the presented wearables. Not only do they affect the players wearing the devices and coaching staff interpreting the data. It rather encompasses whole sports organizations that are required to create opportunities for new marketing products and could in general “re-adjust organizational administrative procedures”.

Furthermore, the availability of data in a contest environment is limited to one’s own team, which allows the analyses of the team tactically, but does not replace the coach’s biased and subjective game observations. For the purpose of tactical analysis it is still questionable, to which extent the current state of wearables assists a coach in a game decision.

### 6.2. Opportunities

A reliability and validity test in a mechanical shaker setting, as conducted by [70], could be used as a template for further intra-, inter-unit and maybe event inter-brand reliability as well as validity testing of the accelerometers on a larger scale. Such a rack could be equipped with all kinds of different tracking devices, or different versions of software on the same device. Eventually, this allows for a somewhat automated and thus standardized testing of the reliability and validity of any kind of wearable.

The built challenge is the current situation of the diverse usage of devices in elite sports teams. As presented in this study in practice sessions, teams employ a range of utilities. However, the inter-unit reliability is not given, nevertheless performances are still taken at face value. Other organizations even employ combined tracker systems’ data such as time–motion analysis, giving rise to club-specific calibration equations. For the scientific community these kinds of constellations are difficult to reproduce, making reliability and validity hard to evaluate.

In general, the metrics help coaches refine their training methods, improve practice quality, prevent injuries and eventually improve the gain of technical skills, physical shape, psychological, and tactical expertise. A proof of this is the highly accepted and widespread application of SSGs in practice. They offer the closest approximation of the physical load in real game situations, which was only possible to measure thanks to the automation of the time–motion analysis. Finally, the more precise the monitoring and assessment of the fatigue or load level of a players is, the better is the coaching staffs’ decision-making to improve practice and game planning. If devices achieve higher accuracy, also in terms of real-time transmission of data to a base station, it could be possible to develop a real-time decision support system as proposed by [159]. The authors introduced a refined version of a decision-making system for coaches that alerts users in case of meaningful strain increase on selected parameters. One of the study’s key elements is the validity of such a metric, as well as the identification of what “meaningful change” means in terms of a given metric. Therefore, more research in terms of reliability and validity of the interpretation of metrics is required.

However, from the current methods of individual and tactical performance analyses, only the individual performance requires this level of reliability and validity. Compared to the tactical measurements such as the team centroid or the inter-team distance, which allows more leeway in terms of data deviation. Given this situation, the general lack of literature and with the assumption that every sport has its own tactical metrics, more research in the area of tactical and decision-making analysis should be conducted.

Finally, there are certain studies presenting the argument that with the progression of miniaturization it is possible to achieve higher accuracy and reliability. There are some contradicting studies, but the latest technological advancements of true 18 Hz GPS sampling show much promise in terms of increased reliability and validity. However, they also seem to create more noise data. Thus, there is need for improved filter algorithms. Another positive aspect of the continuous miniaturization is the eventual introduction of completely new devices to the roster of wearables, such as the muscle oximeter. Not much research has been conducted on this technology in the area of sports exercise, but it already shows auspicious potential to achieve a non-intrusive internal load measurement.

### 6.3. Future Directions

The progression of miniaturization will heavily influence the success of future devices. Compiling more accurate data allows for more research, which is necessary as machine learning algorithms employed for the calculation of individual performance metrics seem to be specific to a sport in question, and thus unreliable when used for others. Hence, there are still many of sports uncovered, especially in the area of invasion sports in either individual, team, or tactical performance measurements. Another very important factor that has not been examined yet is the player’s decision-making. With the complete information in, for example, a practice session, every situation can be recreated and simulated which could allow for the quantification of a player’s decision-making.

Furthermore, there is an interruptive technology shift in the mobile market: the 5G network. The new 5G network is supposed to offer bandwidth of up to regular wireless down- and upload speeds. This new technology affects not all the wearable devices on the market, as some of them already transfer the data via wireless networks. Nonetheless, it would loosen the restrictions of having a local wireless network at the location of data gathering. This could potentially allow for the use of all devices in opposing stadia, as the bandwidth restriction is no longer relevant. The heavier impact, however, is on the shift of the location of processing power. As 5G allows for more data to be transported, the wearables’ data no longer needs to be compressed or aggregated on device. There are, for example, currently some providers that sample the accelerometer and magnetometer at 1 kHz, but provide the data only at 100 Hz. It is unknown to the authors, why this is the case. If the communication bandwidth, on-device processing power, or a combination thereof is the limiting factor of a wearable, the 5G could potentially heavily improve accuracy and reliability of the sensor data. Because the devices can start to provide the originally sampled data at 1 kHz and be processed by unrestricted and dedicated computers. Another impact could be an increase of antennas to receive more precise spatiotemporal data, offering a real alternative to existing GP Systems. Albeit, this is likely to be proprietary data, which is owned and sold by telecommunication enterprises.

Lastly, there are already traceable soccer balls or RFID tags woven into jerseys. The traceable ball allows for a more detailed understanding of motivation of players and their decision-making on the pitch. Increasing further the potential to develop practice instruments to refine a player’s situational awareness. The RFID tags in jerseys as well as the traceable ball facilitate coaches to collect data not only in controlled experimental setups, but also in championship contests, with high accuracy and reliability. Furthermore, manufacturers of the sports tracking devices seemed to couple their vests with further technology and combine their offers of wearable and vest - again to compensate for each device’s shortcomings. The vests are located where the wearables are usually carried, which means they cover the human heart and thus offer a perfect location to sample the heart rate. The wearable on the other hand offers battery and transmission power, rendering an additional heart rate strap redundant and thus improving convenience and potentially add heart rate measuring capabilities to any device.

## 7. Conclusions

This review detailed the current standing of science on wearables. This encompasses their inherent instruments and methods on data gathering for performance analysis, the derived performance metrics, their impact on measurement of team tactics, as well as the environments in which these analyses are conducted. There is still plenty of ongoing debate about reliability and validity of the data generated by the devices. Especially in the domain of highly dynamic, non-linear accelerations, which is predominantly the case in invasion sports. Hence, the wearables, their provided insight metrics and derived interpretations of coaching staff are at least questionable. However, the potential given by the underlying data eventually allows for the support or eventual substitution of qualitative performance measurements with quantitative methods. This encloses instant derivations of load metrics, an evaluation of the state of mind by evaluating the decision-making, the analysis of tactical settings, the prevention of injuries, and the improvement of the performance in general, in practice games as well as in competitive ones. Combining all these factors enables an all in one overarching decision support system that tracks physical, mental, and tactical efficacy aspect of a game or practice.

## Figures and Tables

**Table 1:** Commercially established wearables, as of December 2019.

Name	Loc. Freq.	Loc. Acc.	Accelerometer	Gyroscope	Magnetometer	Heart Rate
SPT GPS 2 ${}^{1}$	10 Hz	+	100 Hz	100 Hz	100 Hz	ext. vest ***
ClearSky T6 ${}^{2}$	100 Hz *	10 cm *	100 Hz ^#^	100 Hz	100 Hz	Polar **
OptimEye S5 ${}^{2}$	10 Hz	50 cm	100 Hz	100 Hz	100 Hz	Polar **
OptimEye X4 ${}^{2}$	10 Hz	100 cm	100 Hz	100 Hz	100 Hz	Polar **
Vector ${}^{2}$	18 Hz	100 cm	100 Hz ^#^	100 Hz	100 Hz	Woven into vest
PlayerTek ${}^{2}$	10 Hz	+	400 Hz	400 Hz	10 Hz	-
Apex ${}^{3}$	18 Hz	+	600 Hz	400 Hz	10 Hz	enabled ****
VXSystem ${}^{4}$	10 Hz	+	104 Hz	18 Hz	18 Hz	Polar **
GPExe Pro 2 ${}^{5}$	18 Hz	+	120 Hz	120 Hz	80 Hz	Polar **
Evo ${}^{6}$	18 Hz	+	100 Hz	-	50 Hz	Polar *
Inmotio LPM	1000 Hz *	3 cm *	-	-	-	Polar **
Kinexon One	60 Hz *	<10 cm *	enabled, +	enabled, +	enabled, +	enabled ****
Johan Sports	enabled, +	+	enabled, +	enabled, +	enabled, +	-
Team Pro ${}^{7}$	10 Hz	+	200 Hz	200 Hz	200 Hz	ext. vest ***

${}^{1}$ Sport Performance Tracking; ${}^{2}$ Catapult Sports; ${}^{3}$ STAT Sports; ${}^{4}$ VXSport; ${}^{5}$ Exelio srl; ${}^{6}$ GPSports; ${}^{7}$ Polar; ^#^ these devices actually sample accelerometer data at 1 kHz, but only provide 100 Hz data; * local positioning measurement; ** has no built in heart rate measurement, but has capabilities to be connected to a Polar device; *** has no built in heart rate measurement, but can be tracked by an extra vest; **** the device is Bluetooth enabled, and can connect to several other bio sensor devices; + no details available; - not supported by the device.
